# Arterial Stiffness as a Surrogate Marker of Cardiovascular Disease and Atherosclerosis in Patients with Vasculitides: A Literature Review

**DOI:** 10.3390/diagnostics13243603

**Published:** 2023-12-05

**Authors:** Konstantinos Triantafyllias, Leif-Erik Thiele, Anna Mandel, Lorenzo Cavagna, Xenofon Baraliakos, George Bertsias, Rebecca Hasseli, Pascal Minnich, Andreas Schwarting

**Affiliations:** 1 Rheumatology Center Rhineland-Palatinate, Kaiser-Wilhelm-Str. 9-11, 55543 Bad Kreuznach, Germanypminnich@students.uni-mainz.de (P.M.);; 2 Department of Internal Medicine I, Division of Rheumatology and Clinical Immunology, University Medical Center, Johannes Gutenberg University, 55131 Mainz, Germany; 3 Department of Internal Medicine, Helios Clinic, 47805 Krefeld, Germany; 4 Department of Rheumatology, University and IRCCS Policlinico S. Matteo Foundation Pavia, 27100 Pavia, Italy; 5 Rheumazentrum Ruhrgebiet Herne, Ruhr-University Bochum, 44649 Herne, Germany; 6 Department of Internal Medicine and Rheumatology, School of Medicine, University of Crete, 71500 Heraklion, Greece; 7 Department of Internal Medicine D, Section of Rheumatology and Clinical Immunology, University Hospital Munster, 48149 Munster, Germany

**Keywords:** cardiovascular disease, arterial stiffness, atherosclerosis, pulse wave velocity, augmentation index, Takayasu’s, giant cell, Kawasaki’s, Behcet’s, granulomatosis with polyangiitis, microscopic polyangiitis

## Abstract

Vasculitis, a group of systemic inflammatory diseases that affect the cardiovascular (CV) system, presents with a variety of clinical manifestations that depend on the size of the affected blood vessels. While some types of vasculitis reveal distinct symptoms, others are characterized by more diffuse and nonspecific presentations that can result in delayed diagnosis and treatment initiation. Interestingly, patients with vasculitides share a significant comorbidity: an elevated CV risk, contributing to increased rates of CV events and mortality. This heightened risk is caused by cumulative inflammatory burden, traditional CV risk factors, medication effects, and reduced physical fitness. Traditional risk assessment tools, commonly used in the general population, frequently underestimate the CV risk in patients with inflammatory rheumatic conditions. Consequently, novel approaches are necessary to stratify the precise CV risk in vasculitis patients. A number of surrogate parameters for CV risk have been investigated, with arterial stiffness emerging as a promising marker. Pulse wave velocity (PWV) is a well-established method for assessing arterial stiffness and predicting CV risk across different populations. Among numerous PWV variants, carotid–femoral PWV (cfPWV) stands out as the most extensively studied and accepted reference standard. It has demonstrated its utility as a surrogate CV parameter both in the general population and in patients with systemic inflammatory rheumatic diseases. In recent years, research has expanded to assess arterial stiffness in systemic rheumatic diseases, such as arthritis, connective tissue diseases, rheumatologic overlap syndromes, and chronic pain disorders, using measurements of PWV and other markers of arterial compliance and elasticity. Despite burgeoning research in rheumatologic diseases, data on CV risk markers in vasculitides remain limited and fragmented. This narrative review aims to provide a comprehensive overview of arterial stiffness as a potential screening marker for CV diseases, atheromatosis, and ultimately CV risk among patients with vasculitides.

## 1. Introduction

The term vasculitis is used to summarize a diverse group of systemic inflammatory diseases impacting the cardiovascular (CV) system [1]. According to the 2012 Chapel Hill consensus conference classification, vasculitides can be classified into various categories depending on the size of the affected vessel, including large, medium, and small vessel vasculitides, as well as vasculitides of varying size [2]. Due to the diverse vessel involvement, vasculitides may show highly variable symptoms and clinical signs, ranging from headaches and temporal pain in patients with giant cell arteritis, to skin, lung, and renal involvement in those with small vessel size vasculitides [3]. Nevertheless, some vasculitic entities might cause diffuse and nonspecific symptoms, which may significantly delay diagnosis and thus treatment initiation. These vasculopathies can affect certain patient groups, (for instance Asian children with Kawasaki disease (KD)), yet all result in chronic and systemic inflammation [1,4].

Patients affected by vasculitides share a major comorbidity: an increased CV risk due to significantly higher rates of CV events and mortality as compared to the general population [5,6,7]. For example, a standardized rate of 2.3-fold higher has been reported for CV mortality in patients with small vessel vasculitis. Additionally, there is a significant association between MPO-ANCA vasculitis and CV death [8]. Reasons for the increased risk in patients with vasculitis and rheumatologic conditions are mainly attributed to the cumulative effects of inflammatory activity during the course of the disease, a higher prevalence of traditional CV risk factors, the effect of medications like glucocorticoids, and impaired physical fitness [9]. However, rheumatic patients are often not recognized and treated for their risk of developing CV disease (CVD), as common stratification systems fail to assess the aforementioned disease-specific high levels of inflammation and cardiovascular burden [10]. 

Traditionally, CV risk assessment in the general population includes evaluating parameters such as age, blood pressure, smoking status, lipid levels, and family history of CVD. In clinical practice, scoring systems like the Framingham risk score, PROCAM, and Systematic Coronary Risk Evaluation (SCORE) of the European Society of Cardiology are well-established traditional tools for assessing CV risk [10,11,12,13]. These methods are both useful and practical for identifying individuals at risk for CVD in the general population. However, these calculators often tend to underestimate CV risk in patients with inflammatory rheumatic conditions. As a result, alternative tools have been investigated in recent years, including the Expanded Cardiovascular Risk Prediction Score, the QRISK-3, and the 1.5-SCORE multiplier, which account for the presence of certain rheumatic conditions [14,15]. However, validation of these CV assessment tools has primarily been performed in patients with prototype rheumatic diseases, including rheumatoid arthritis (RA) and systemic lupus erythematosus (SLE) [14,15], therefore CV risk assessment in rarer rheumatologic conditions such as vasculitis can be challenging.

Consequently, there is a need for additional biomarkers to stratify CV risk in patients with vasculitides, and different CV surrogate parameters have been examined in this regard [16,17]. One of the most important and well-studied surrogate assessment markers of CV risk is arterial stiffness [18]. As thoroughly discussed in our previous review on the diagnostic value of arterial stiffness in patients with arthritides and connective tissue diseases [19], a plethora of methods can be employed to assess arterial stiffness. These include the augmentation index (AIx) [20], the cardio-ankle vascular index (CAVI) [21], and the β-stiffness index. Nonetheless, one of the most well-examined methods in the general population is pulse wave velocity (PWV) [22]. 

Several studies, including various meta-analyses, have demonstrated that arterial stiffness, as measured by PWV, can accurately predict CV risk in both high and low CV risk cohorts from the general population (23-25). In a comprehensive meta-analysis involving 17 studies with 15,877 subjects, Vlachopoulos et al. found that pooled relative risks (RR) of CV mortality were significantly higher for subjects with high aortic PWV, compared to those having low PWV values (RR: 2.02, 95% CI: 1.68 to 2.42) [23]. The RR of CV mortality by increased aortic PWV was especially elevated in high-risk populations (RR: 2.48; 95% CI: 1.94 to 3.18), but also in cohorts with apparently low risk (RR: 1.68, 95% CI: 1.41 to 2.01; *p* = 0.013) [23]. In 2017, Ohkuma et al. came across similar results by examining another oscillometric marker of arterial stiffness (brachial–ankle PWV; baPWV) in 14,673 Japanese participants [24]. Here it was shown that higher baPWV values were significantly associated with a higher risk of CVD even after adjustments for traditional risk factors (*p* for trend <0.001). Furthermore, in their examination of 17,635 subjects, Ben-Shlomo et al. in 2013 found an increased HR of 1.45 (95% CI: 1.30 to 1.61; *p* < 0.001) for CVD in patients with an elevated aortic PWV [25]. Sequí-Domínguez et al. conducted a meta-analysis of six studies to examine the predictive capability of cfPWV for CV mortality. The results showed that cfPWV is a reliable predictor with a diagnostic odds ratio (dOR) value of 11.23 (95% CI, 7.29–1.29, *n* = 3170) [26].

Of all PWV variants, cfPWV is the most adequately examined and therefore described as a reference standard. There is strong evidence supporting its utilization as a surrogate CV parameter in both the general population and in patients with systemic inflammatory rheumatic diseases [27]. In recent years, our research group and others have examined arterial stiffness of the aortic vasculature in the context of several systemic rheumatic diseases, such as RA [28], mixed connective tissue disease [29], SLE [30], antisynthetase syndrome [31], systemic sclerosis [32], and chronic pain syndromes [33], using cfPWV as an assessment tool. 

Even though there has been increasing research in the field of rheumatologic diseases, data focusing on CV risk markers in patients with vasculitides are still scarce and have only been partially presented. Thus, this review will provide an overview of the value of arterial stiffness as a screening marker for CV diseases, atheromatosis, and ultimately CV risk in a wide spectrum of vasculitic entities. 

## 2. Methods

**Figure 1:**
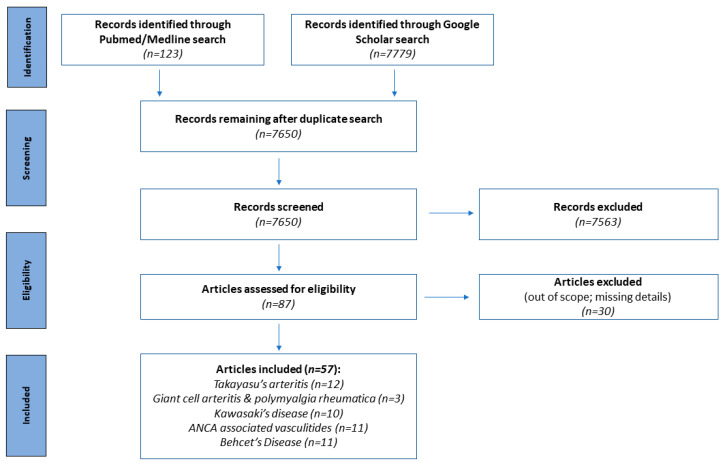
PRISMA flow diagram of the article screening, inclusion, and exclusion process [34].

For this narrative review, a search strategy was applied to enable us to identify the most important literature on the topic. An exhaustive search was performed on PubMed/Medline and Google Scholar using a combination of keywords according to the Boolean operators: “arterial stiffness” AND (“Takayasu’s arteritis” OR “giant cell arteritis” OR “polymyalgia rheumatica” OR “Kawasaki’s disease” OR “granulomatosis with polyangiitis” OR “eosinophilic granulomatosis with polyangiitis” OR “microscopic polyangiitis” OR “Behcet’s disease”) (3305); as well as (“pulse wave velocity”) AND (“Takayasu’s arteritis” OR “giant cell arteritis” OR “polymyalgia rheumatica” OR “Kawasaki’s disease” OR “granulomatosis with polyangiitis” OR “eosinophilic granulomatosis with polyangiitis” OR “microscopic polyangiitis” OR “Behcet’s disease”) (5562). This produced a total of 8867 publications. Limiting the results to publications from the past two decades left us with 7902 articles across all keyword combinations. We then manually excluded irrelevant and duplicated papers, as well as papers without full text, by skimming over the titles and, where necessary, the abstracts (Figure 1).

## 3. Basic Principles of Arterial Stiffness Measurements

In our previous review work published in this journal, discussing the diagnostic value of arterial stiffness in patients with arthritides and connective tissue diseases, we have thoroughly described the definition and the main pathogenetic mechanisms of arterial stiffness [19]. Moreover, we have discussed the most important assessment methods of arterial stiffness and their calculation formulas [19]. A summary of these points is now provided in Table 1 and Table 2. 

## 4. Aortic/Arterial Stiffness as a Surrogate CV Marker in Patients with Systemic Vasculitis

### 4.1. Large Vessel Vasculitides

#### 4.1.1. Takayasu’s Arteritis

**Figure 2:**
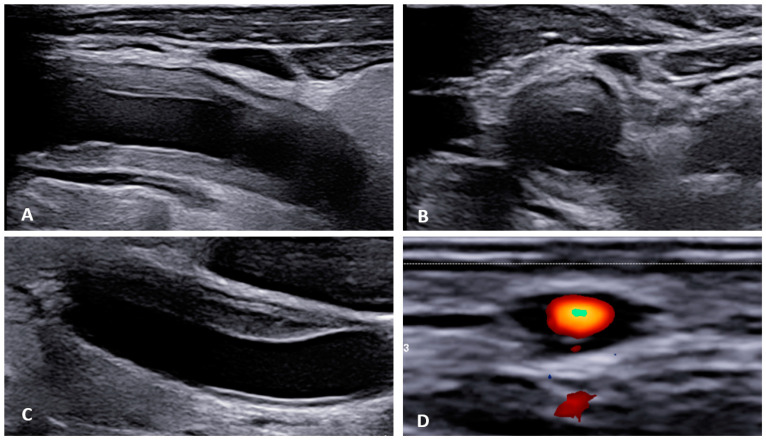
Ultrasonographic findings in patients with large vessel vasculitis: (**A**,**B**): B-mode US with the finding of concentric carotid intima-media thickening in a patient with Takayasu’s arteritis ((**A**) longitudinal plane, (**B**) transversal plane). (**C**). B-mode US: carotid intima-media thickening in a patient with giant cell arteritis. (**D**): Power Doppler US in a patient with temporal arteritis (typical “HALO” sign). Courtesy of Dr. Konstantinos Triantafyllias, Rheumatology Center, Rhineland-Palatinate.

Takayasu’s arteritis (TA) is a rare chronic inflammatory granulomatous disease of larger vessels, such as the aorta and its branches [36]. Its origin remains unclear, and patients are primarily young females (<40 years) and children [37]. TA exhibits unspecific clinical symptoms, such as fever, joint tenderness, and headaches, as well as specific vascular symptoms, including Raynaud’s syndrome, syncopes, or erythema nodosum [37]. Moreover, CV complications such as myocardial infarction, stroke, or peripheral artery disease are not uncommon [37]. Imaging of large vessels is paramount for the diagnosis of TA (Figure 2A,B).

Several studies over the past years have examined arterial stiffness in the context of TA [38,39,40,41,42,43,44,45,46,47]. The majority of these studies employed a case–control design with a small number of subjects. However, the findings suggest that TA patients exhibit increased arterial stiffness compared to healthy controls. For instance, Salles Rosa Neto et al. conducted a study assessing cfPWV in 27 Brazilian women with TA and 27 female healthy controls with comparable age, blood pressure, height, and weight [38]. The results revealed significantly higher cfPWV values in TA patients compared to controls (9.77 ± 3.49 vs. 7.83 ± 1.06 m/s; *p* = 0.009) [38]. Moreover, in the same study, significantly increased pulse pressures were found in the TA group (54 ± 22 vs. 40 ± 9 mmHg; *p* = 0.005) [38]. 

Similarly, in a follow-up study using echocardiography with pulse wave Doppler to assess carotid–femoral arterial stiffness, Yang et al. found higher cfPWV values in 25 TA patients when compared to 25 strictly matched healthy controls (8.37 ± 2.23 vs. 6.46 ± 1.15 m/s; *p* < 0.001), [39]. There was low intra- and inter-observer variability observed during the evaluation of 15 randomly selected TA patients and 15 healthy controls, demonstrating the method’s satisfactory reproducibility [39]. 

Raninen et al. found also statistically significant increased carotid (*n* = 13) and femoral (*n* = 16) artery stiffness values in TA patients during ultrasound examinations, using different elastic moduli (Peterson’s elastic modulus: *p* = 0.019, Young’s elastic modulus: *p* = 0.013 and beta stiffness constant: *p* = 0.004) [40]. 

Regarding predictors of arterial stiffness in patients with TA, there is evidence that elevated NT-pro-BNP (a known marker of heart failure) may be associated with stiffer arteries, as shown in two studies [41,42]. In particular, according to the cross-sectional study of Liu et al. including 72 TA patients, baPWV could be predicted by NT-pro-BNP (*p* = 0.036), next to mean blood pressure (*p* < 0.001) and age (*p* = 0.002) [41]. Comparable assessments were also provided by Tombetti et al., where NT-pro-BNP was described as a promising biomarker for predicting organ damage in patients with large vessel vasculitis (LVV), such as TA [42]. 

In another small study of seven pediatric patients with TA, Grotenhuis et al. examined the stiffness of two different parts of the arterial tree (carotid–radial and carotid–femoral) by PWV [43]. It was found that cfPWV (8.3 ± 1.9 m/s) and crPWV (8.1 ± 1.8 m/s) were statistically significantly higher in the TA group compared to the control group (5.1 ± 0.8 m/s, *p* < 0.01, and 6.4 ± 0.6 m/s, *p* = 0.03, respectively) [43]. However, while crPWV correlated with disease activity, aortic stiffness (cfPWV) did not [43]. Of course, the number of patients included in this study was very low, and thus the results should be interpreted with caution.

Included patients were shown to have impaired left ventricular function, but this appeared to be independent of acute disease activity [43]. Interestingly, the lack of association between increased arterial stiffness and acute systemic inflammation has also been described by different researchers, who found increased arterial stiffness values even in patients perceived to be in remission [38,44]. On the contrary, in the study of 67 subjects with TA, Wang et al. found baPWV to be significantly higher in patients with active disease than in patients in remission (*p* = 0.04). Nevertheless, there was no correlation found between baPWV and CRP or ESR levels in the entire TA group (both; *p* > 0.05) [45].

Another study revealed that patients with TA not only show an elevated risk for CV disease but also seem to remain at risk even after therapeutic intervention with a drug-eluting stent (DES) [46]. In this specific exploration, which included 48 TA patients, 12.5% of the treated subjects experienced major adverse cardiac events (MACE), defined as all-cause death, nonfatal myocardial infarction, or nonfatal target vessel revascularization, during an average follow-up period of 25.6 months (ranging from 9.0 months to 68.0 months) [46]. Moreover, during the same period, restenosis occurred in 48/73 treated lesions. A baPWV value of ≥17 m/s was identified as a possible independent predictor of DES restenosis (OR 5.50, 95%CI 2.1–16.6, *p* = 0.008). In addition, the same baPWV cut-off (≥17 m/s) was linked to a 3.4 times greater risk for MACE [46]. He et al. determined a similar cut-off value for baPWV in risk evaluation of general CV events (16.26 m/s). However, their study yielded low sensitivity (45.9%) and a specificity of 83.7% [47]. 

Altogether, TA appears to be associated with increased arterial stiffness, increasing the susceptibility of TA patients to CV events. PWV may be suitable as a screening method to identify patients at risk. More research into supplementary markers such as NT-pro-BNP and sensible cut-off values of baPWV is needed.

#### 4.1.2. Giant Cell Arteritis/Polymyalgia Rheumatica

**Figure 3:**
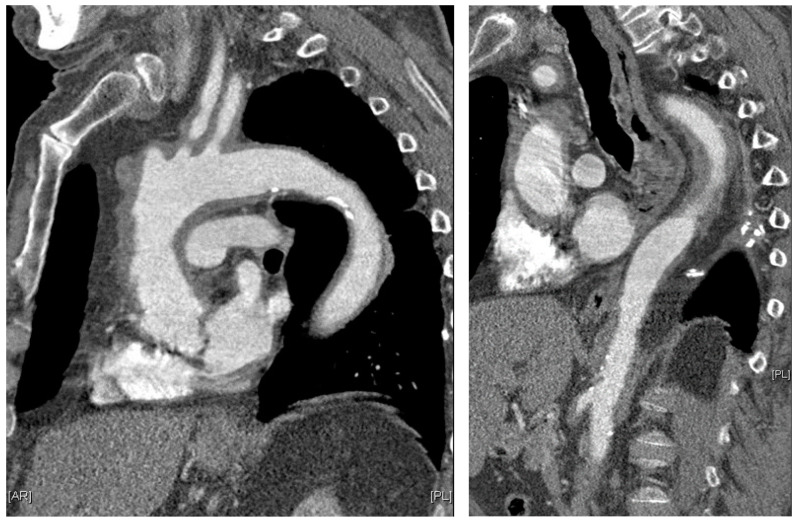
Parasagittal thoracic and sagittal abdominal MPR images on contrast-enhanced computed tomography: pronounced, circular, long-stretch vessel wall thickening of the aorta and its branches in giant cell arteritis. Courtesy of Dr. Corinna Schorn, Rheumatology Center, Rhineland-Palatinate.

**Figure 4:**
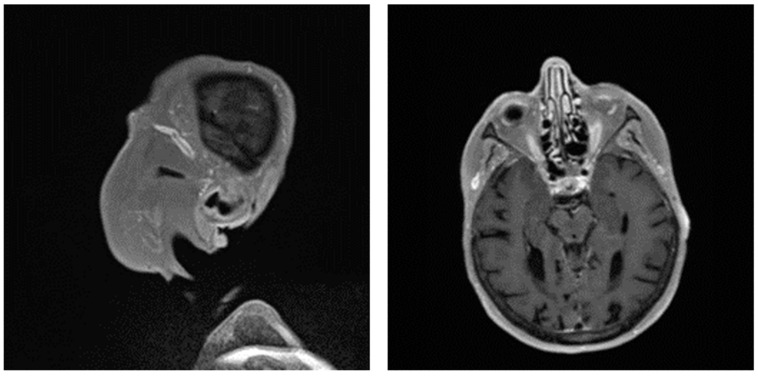
Pronounced wall thickening of the temporal arteries, as depicted on black blood magnet resonance imaging sequences (T1 space). (Courtesy of Dr. Corinna Schorn, Rheumatology Center, Rhineland-Palatinate).

Giant cell arteritis (GCA) affects typically large- and medium-sized arteries, including the carotid arteries and their extracranial branches, as well as the aorta and its branches [48]. Symptoms of GCA include headache, jaw claudication, tenderness of the temporal artery, and low-grade fever [49]. Typical complications of GCA include acute loss of vision and aortic aneurysms, which in turn can lead to more severe complications like stroke or aortic dissection [49,50]. GCA is frequently linked with polymyalgia rheumatica (PMR), characterized by muscle pain and bilateral morning stiffness in the proximal extremities. At a microscopic level, giant cells infiltrate the vascular wall, leading to an occlusion of the affected blood vessels and thus to ischemia of the tissue to be supplied [51]. The diagnosis of PMR/GCA is usually based on clinical presentation, imaging, temporal artery biopsy (Figure 2C,D, Figure 3 and Figure 4), and the ACR/EULAR classification criteria [49,52]. 

Data on arterial stiffness in patients with GCA and/or PMR are limited to a small number of studies [53,54,55]. One study of 39 PMR patients found that they exhibited higher arterial stiffness, as measured by PWV, compared to healthy controls matched for sex, age, and blood pressure (12.4 ± 4 m/s vs. 10.2 ± 2 m/s, *p* < 0.01) [53]. Another study by Pieringer et al., which compared 13 newly diagnosed PMR patients to 30 age- and sex-matched controls, found that AIx tended to be higher in the PMR group [54]. Aortic PWV was significantly associated with age, male sex, left systolic and diastolic blood pressure, right diastolic blood pressure, and CRP in a cohort of 77 newly diagnosed patients with GCA, PMR, and an overlap between GCA/PMR [55].

Regarding the effects of immunosuppressive medication, Schillaci et al. compared cfPWV values of GCA patients with those of matched controls at baseline and after 4 weeks of treatment with glucocorticoids [53]. A significant decrease under glucocorticoid therapy (from 11.8 ± 3 m/s to 10.5 ± 3 m/s, *p* = 0.015) could be observed. A more recent study (2021) by Emamifar et al. monitored the arterial stiffness parameters of 77 patients undergoing oral glucocorticoid treatment for a time period of 40 weeks [55]. The study showed a decrease in aortic PWV at week 16 (*p* = 0.010), which persisted at week 28 (*p* = 0.002) and week 40 (*p* < 0.001) compared to baseline values. However, no significant decrease in the PWV values was observed beyond week 16. Interestingly, AIx (and normalized AIx for 75 pulses/minute; AIx@75) did not significantly differ from baseline values during the follow-up period in this study. On the other hand, in the studies of Schillaci et al. and Pieringer et al., AIx was significantly decreased after treatment with glucocorticoids [53,54]. 

In summary, based on the limited available evidence, arterial stiffness seems to be significantly increased in patients with PMR and/or GCA. Additionally, anti-inflammatory treatment may result in a reduction of PWV and thus decrease CV risk. However, studies with higher counts of patients and longitudinal designs are necessary to confirm these findings.

### 4.2. Medium Vessel Vasculitides

#### Kawasaki’s Disease

KD is a necrotizing vasculitis of the medium and smaller vessels that often involves the coronary arteries, resulting in aneurysms, that can persist even after clinical remission [56,57,58]. It predominantly affects young children, particularly those of Japanese descent, and presents with antibiotic-resistant fever, conjunctivitis, rashes, and cervical lymphadenopathy. KD can often result in fatality if left untreated, mainly due to CV manifestations. These may include myocardial infarction in the acute phases of the disease or chronic damage that persists for years after its apparent recovery [59].

KD patients are often categorized into two phenotypical subgroups based on whether they present with coronary involvement (mostly aneurysms) (KD+) or not (KD-). In general, most of the studies indicate that KD is associated with increased arterial stiffness, as observed through measurements such as PWV [56,57,58,60], echocardiography [61], or AIx [62]. Furthermore, KD+ patients have been shown to have significantly higher baPWV [57,58,60,61], carotid artery index [57], and stiffness index [58], compared to their KD- counterparts. 

In a relatively large study conducted by Ooyanagi et al., 90 KD patients were compared with 119 control subjects [56]. Hereby, it was found that when a cut-off point of ≥120% of the normal predicted PWV was set, patients with a history of KD had higher PWV values than control subjects [56]. However, no significant difference was found between the ABI values of the two groups [56]. 

On the other hand, a recent cross-sectional case–control study consisting of 60 patients found no significant PWV differences between the included patient and control group [63]. Other studies also reported slightly, but insignificantly increased values for characteristic impedance (Zc), input impedance (Zi), elastic pressure–strain modulus (Ep), and beta stiffness index [61], but not for cardio ankle vascular index (CAVI) [60]. These contradicting findings may be attributed to significant diversity in study design when assessing arterial stiffness in KD, as evidenced by Patra et al. (2022) in a recent systematic review and meta-analysis of 49 case–control studies with 2714 cases and 2118 controls (0.35, 95% CI: 0.11–0.59) [64]. 

Interestingly, KD patients appear to experience accelerated atherosclerosis, as evidenced by significantly lower ankle–brachial indices [56] and significantly higher intima-media thickness of the carotid artery (cIMT) between patients and controls [57,58,65]. Cheung et al. and Dietz et al. observed significant differences in cIMT between KD+ and KD- patients but were unable to demonstrate significant differences between KD- patients and controls [58,65]. Thus, Dietz et al. pointed out a greater need for rigorous follow-up measures among controls in KD+ than KD- patients [65]. An additional case–control study by Chen et al. found a higher aortic IMT (0.53 mm (95%CI: 0.51–0.56) vs. 0.49 mm (95%CI: 0.47–0.52), *p* = 0.04) in KD patients in comparison to control subjects [63].

Disease duration has been proposed as a possible predictor of increased arterial stiffness in KD, as longer duration of disease correlated positively with higher PWV in an exploration including 42 KD patients (4.95 m/s vs. 3.70 m/s, *p* = 0.0008) [61].

In conclusion, KD patients seem to be at a higher risk for abnormal arterial stiffness and atherosclerosis development compared to healthy controls, and chronic damage in the context of the disease may predict these pathologies. However, the high heterogeneity of studies should also be taken into account, making the extraction of certain conclusions challenging. 

### 4.3. Small Vessel Vasculitides

#### ANCA Associated

ANCA-associated vasculitis (AAV) is a term covering several entities including granulomatosis with polyangiitis (GPA), eosinophilic granulomatosis with polyangiitis (EGPA), and microscopic polyangiitis (MPA) [66]. 

GPA is a necrotizing vasculitis affecting predominantly medium and small vessels. Patients typically present with progressive development of symptoms. Early involvement in the disease leads to ulcers and non-caseating granulomas in the upper respiratory tract and lungs, which eventually progress to involve the kidneys in over 75% of cases [67]. A 10-year survival rate of more than 80% is possible with optimal treatment; however, organ damage can worsen prognosis significantly [68]. EGPA is comparable to GPA but is characterized by eosinophilic infiltrations of extravascular tissue and its association with asthma (>90%). While kidney involvement occurs less frequently (20%) in EGPA, there is a high incidence of cardiac involvement with myocardial infarction and heart failure being the primary causes of death [69].

Both GPA and EGPA are associated with a significantly increased risk for CV events. In a meta-analysis of Houben et al., seven studies with about 14,000 patients were included. These patients exhibited a relative risk of 1.65 (95% CI: 1.23, 2.22) for experiencing a CV event compared to the general population [70]. Several studies have shown that AAV patients with increased PWV are at a higher risk of experiencing CV events, emphasizing the significance of early detection and prevention care [71,72,73,74]. Other markers of arterial status yielded inconsistent results in different studies. For instance, Pacholczak et al. found a significant difference in an endothelial function marker (flow-mediated dilatation; FMD, *p* < 0.011) without finding statistical differences in AIx among 44 AAV patients and 53 control subjects [75]. Contrary to expectations, FMD% was inversely correlated to smoking status (packs/years, *β* = −0.33 (95% CI: −0.44 to −0.12)) in AAV [75]. 

Regarding carotid atheromathosis, Gonzalez-Suarez et al. found an increase in cIMT that was independent of traditional CV risk factors (*n* = 23) [76]; furthermore, cIMT was correlated with kidney involvement [75] and the number of disease relapses [77]. Interestingly, Pacholczak et al. found no difference in cIMT in AAV without kidney involvement when compared to healthy controls (*n* = 44) [75]. 

An increase in immune cells in the context of systemic inflammatory disease may be a contributing factor to the stiffening of arteries in AAV. Recent studies have concentrated on CD4+CD28null cells, a specific category of T-cells, as a potential influence in the pathogenesis of premature arterial stiffening and accelerated atherosclerosis [78,79]. These cells were found to be associated with a history of CMV infection but not with an accelerated atheromatosis in a study of 40 individuals with AAV. The pathophysiological mechanisms possibly involve cytotoxic effects on endothelial cells and a destabilizing impact on atherosclerotic plaques. Additionally, CD4+CD28null seems to be more prevalent in RA patients with higher IMT [72].

Inflammation generally appears to be the driving factor in atherosclerosis and thus arterial stiffness. Accordingly, AAV frequently shows markedly elevated CRP levels, even in remission [72,80], along with IL-6 [75], which in turn are positively linked to an escalation in markers for vascular impairment such as VCAM-1 and thrombomodulin (15.9% higher levels of VCAM-1, *p* = 0.01; 50.9% increase in thrombomodulin concentration, *p* < 0.001; *n* = 44, in peripheral blood tests compared to healthy individuals) [75]. The increase in VCAM-1 has also been shown to correlate with a higher CD4+CD28null count [79] and with kidney involvement [75]. However, disease damage, and thus the effects of chronic cumulative inflammation, was not found to correlate with IMT in a small case–control study examining 46 GPA patients (OR 0.88, *p* = 0.48, *n* = 46) [77]. 

To summarize, patients with small vessel vasculitides have a higher prevalence of CVD and surrogate markers of CV risk may assist risk stratification. Thus, the generation of data on the predictive value of these markers is of great importance. 

### 4.4. Varying Size Vasculitides

#### Behcet’s Disease

Behcet’s disease (BD) is a chronic systemic inflammatory vasculitis characterized by diffuse genital and oral ulcers, rooted in chronic vasculitis of varying size vessels [81]. While the pathogenesis of BD remains unclear, studies suggest that infection-related triggers may cause the disease in genetically predisposed patients [82]. Patients of Middle Eastern descent are typically affected. In addition to pathognomonic ulcers, other significant symptoms include uveitis, arthralgia, and fatigue [83]. 

A 2017 meta-analysis by Upala et al. (*n* = 303) found that BD patients had significantly greater cfPWV compared to healthy controls (pooled mean difference (MD) = 0.74; 95%, CI: 0.28–1.20, *p* = 0.002) [84]. This is in line with the results of other studies linking BD to increased PWV [85,86,87]. Additionally, further arterial stiffness parameters have been found to have higher values based on several examinations. These parameters include stiffness index (5.98 ± 1.17 m/s vs. 5.34 ± 0.70 m/s; *p* = 0.021, *n* = 96) [88,89], distensibility coefficient (DC), incremental elastic modulus (Einc) (Ree et al.; *n* = 41, DC: *p* = 0.021, beta: *p* = 0.007, Einc: *p* = 0.008), and shear wave coefficient (Alis et al.; *n* = 34, *p* = 0.001 mean right, *p* = 0.003 mean left, respectively) [90,91]. All of these markers were significantly increased in BD, providing additional evidence linking this vasculitis to increased arterial stiffness.

Data regarding the relationships between arterial stiffness, acute inflammatory activity, and chronic BD-associated damage, as assessed by disease duration, are conflicting, possibly due to heterogeneous study designs, small patient sample sizes, and diverse measures of arterial stiffness [47,49,55,87,89,92]. Further studies on the topic focused on the establishment of potential predictors of arterial stiffness: Rhee et al., for instance, found a correlation between arterial stiffness parameters and age at onset, as well as with peripheral arthritis in the context of the disease [91]. Regarding the effects of medication, in the study of Protogerou et al., the use of glucocorticoids seemed to result in a lowering of AIx but not PWV (21 ± 14% vs. 12 ± 14%, *p* < 0.05, *n* = 74) [88]. 

The data on atherosclerosis, as measured through cIMT, are inconclusive. Some studies did not show a significant increase in cIMT in patients with BD (0.52 ± 0.09 mm vs. 0.52 ± 0.06 mm, *p* = 0.811, *n* = 41) [85,91], while others found the opposite trend (*n* = 34; right side: 0.5 ± 0.11 mm vs. 0.4 ± 0.07 mm; left side: 0.5 ± 0.14 mm vs. 0.41 ± 0.11 mm; *p* = 0.001 and *p* = 0.003, respectively) [90]. In the study by Yildirim et al., cIMT appeared to correlate with the duration of the disease [86].

Patients with BD exhibit varying degrees of arterial stiffness, depending on the disease phenotype. Increased arterial stiffness is also observed in patients with Behçet’s disease (BD), and this phenomenon appears to be influenced by the type of BD: mucocutaneous BD patients exhibit higher carotid–femoral pulse wave velocity (cfPWV) despite having a protective lipid profile [85]. Conversely, systemic BD patients display even higher cfPWV values than mucocutaneous BD patients (8.48 ± 1.14 m/s vs. 7.53 ± 1.40 m/s, *p* = 0.017) and show elevated levels of LDL, total cholesterol, and triglycerides [85]. Triglycerides were identified as a significant contributing factor to increased PWV levels, according to linear regression analysis (*p* = 0.001, *n* = 23). Of note, Caldas et al. were the first to report this significant difference [85].

Endothelial dysfunction appears to play a crucial role in the development of arterial stiffness in BD patients [35]. Specifically, BD patients exhibit significantly reduced endothelial-dependent vasodilation compared to healthy controls, a difference observed independent of vascular involvement. Although endothelial-independent vasodilation was also lower in BD patients, this difference did not reach statistical significance. Homocysteine levels, which influence vasoactivity via NO were significantly elevated in BD patients compared to healthy controls (plasma homocysteine levels 13 ± 6 micromol/L vs. 9 ± 3 micromol/L, *p* = 0.001, *n* = 65). Patients with vascular involvement among BD patients had the highest homocysteine levels compared to reference groups (15 ± 7 micromol/L vs. 12 ± 4 micromol/L, *p* = 0.03, *n* = 27) [93]. Moreover, BD is believed to have an unfavorable impact on blood pressure variability. A considerable portion of BD patients do not exhibit the conventional physiological drop in blood pressure during the nighttime, a phenomenon viewed as a predictor of increased cardiovascular risk. 

A summary of the most important studies included in our work is provided in Table 3.

## 5. Discussion

In vasculitides, CVD has been established as one of the leading causes of premature mortality, increased morbidity, and impairment of quality of life. Although conventional CV-risk tools exist for the general population, they only consider traditional risk factors such as SBP, BMI, or dyslipidemia and thus may be suboptimal in evaluating the CV status of patients with systemic inflammatory rheumatic diseases [31].

Numerous studies of various diseases indicate that inflammation increases the risk of cardiovascular disease. Although the exact pathomechanisms of increased aortic stiffness are not yet completely clear, systemic inflammation negatively affects arterial status by accelerating atherosclerosis, causing endothelial dysfunction, and ultimately leading to higher rates of CV events and mortality [94]. An assessment of aortic stiffness, including measurement of PWV, AIx, or β-stiffness index might serve as a non-invasive, relatively easy-to-use, and low-cost tool in the quest of pinpointing patients at risk [95]. 

Thus, this review work aimed to present some of the studies that have examined the diagnostic value of arterial stiffness as a surrogate marker of CVD in patients with systemic vasculitides. A strength of the present work is the fact that research studies evaluating multiple vasculitic entities, regardless of the vessel size, such as small vessel vasculitides, GCA, KD, and BD, were included. Moreover, this work is one of the very few to collectively present data on CV surrogate markers in patients with systemic vasculitides. 

**Figure 5:**
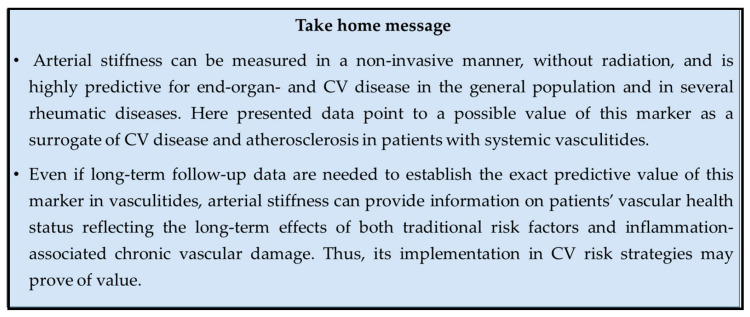
The take-home message of the work.

There are, however, also some limitations to this work. First, we have examined only articles written in the English language. This and other factors that could be associated with publication bias (i.e., more published studies with positive results) should be taken into account. Secondly, a high level of study design heterogeneity, significant variability in assessed arterial stiffness parameters, and a predominance of cross-sectional studies with a low number of participants could be identified. This can complicate the ability to draw definitive conclusions about the diagnostic value of arterial stiffness and CV risk. Long-term studies with follow-up assessments and thorough evaluation of links between these markers and hard CV endpoints are therefore warranted. Nevertheless, arterial stiffness enables an evaluation of the arterial tree and its elastic properties, providing additional information regarding CV burden and CVD [96] (Figure 5). Furthermore, it can serve as a screening tool for asymptomatic patients and reflect the long-term effects of chronic inflammation on the CV system [19]. 

## Figures and Tables

**Table 1:** Main assessment methods of arterial stiffness (adapted from [19]).

Assessment Methods of Arterial Stiffness			
Marker	Methodology	Clinical Association	Calculation
Pulse wave velocity (PWV)	Oscillometric marker	Reflects the speed of pulse waves traveling between two sites of the arterial tree	Calculated by dividing the distance between these sites by the pressure wave transit time (∆s/∆t)
Augmentation index (AIx)	Marker based on applanation tonometry	Represents the supplementary systolic blood pressure increase, which is mainly caused by wave reflections	Calculated as the ratio between (1) the difference between peak systolic pressure and the shoulder of the ascending part of the blood pressure curve, and (2) the pulse pressure
Aortic stiffness index (AoSI)	Doppler echocardiography marker	Measures aortic stiffness 3 cm above the aortic valve	Calculated by the following formula: AoSI = ln(SBP/DBP)/(AoS − AoD)/AoD (where SBP = systolic blood pressure and DBP = diastolic blood pressure)
β-stiffness index	Elastic parameter usually assessed by carotid ultrasound, coupled with applanation tonometry data	Indirectly measures the change in the internal luminal diameter of the carotid artery in the radial direction	Calculated by the formula: β-stiffness index = ln(SBP/DBP)/[(Ds − Dd)/Dd]
Aortic distensibility	Echocardiographic measure	Reflects aortic stiffness	Calculated by the formula: aortic distensibility = (2×)/(AoS − AoD)/AoD/(SBP − DBP)
Cardio-ankle vascular index (CAVI)	A marker that is associated with PWV, being however less dependent on arterial pressure	Reflects stiffness of the cardio-ankle part of the arterial tree	Calculated by the formula CAVI = a[(2ρ/(SBP − DBP)) × ln(SBP/DBP) × PWV2 ] + b (a and b are constants; ρ = blood density, PWV: brachial–ankle PWV)

PWV: pulse wave velocity; AIx: augmentation index; AoSI: aortic stiffness index; SBP: systolic blood pressure; DBP: diastolic blood pressure; SD: end-systolic diameter; DD: end-diastolic diameter; AoS: aortic systolic diameter; AoD: aortic diastolic diameter; CAVI: cardio-ankle vascular index; ρ: blood density; a and b: constants.

**Table 2:** Definition and basic pathogenetic mechanisms of arterial stiffness (adapted from [19,35]).

Definition of Arterial Stiffness	Loss of Arterial Compliance. Association with CVD Due to Systolic Hypertension and Heightened Pulse Pressure
Basic Pathogenetic Mechanisms	
Arterial wall (extracellular matrix) composition changes	Fragmentation of elastin fibers, deposition of collagen (stiff wall material), and cross-linking of collagen molecules.
Activation of renin–angiotensin system	Changes in the arterial wall by the proliferation of vascular smooth cells, inflammatory activity, and collagen increase.
Effects of chronic arterial hypertension	Stretching of the arterial wall by pulse pressure, stiffer vessel ‘‘appearance‘‘ during examination.
Functional arterial stiffening	Endothelial dysfunction: reduced NO synthesis, activity, and content.
Further pathophysiological mechanisms	Acceleration of atherosclerosis, effects of autoantibodies, metabolic components, and further traditional CV risk factors.

CV cardiovascular; CVD cardiovascular disease.

**Table 3:** Studies examining arterial stiffness as a cardiovascular surrogate.

Reference	Vasculitis	Marker(s) of Arterial Stiffness	Study Population	Statistical Analysis
Salles Rosa Neto et al. [38]	TA	cfPWV and AIx	N = 27	cfPWV higher in TA than in HC (9.77 ± 3.49 vs. 7.83 ± 1.06 m/s; *p* = 0.009)
Yang Y et al. [39]	TA	cfPWV via echo	N = 25	cfPWV higher in TA than in HC (8.37 ± 2.23 vs. 6.46 ± 1.15 m/s; *p* < 0.001)
Raninen RO et all. [40]	TA	Carotid and femoral ultrasound	*n* = 29	Ep and YM were significantly higher than HC: carotid: *p* = 0.019 and 0.013/femoral: *p* = 0.005 and 0.039. Carotid stiffness index is also higher than in HC (*p* = 0.004).
Liu Q et al. [41]	TA	baPWV	*n* = 72	CV risk markers significantly higher in the high baPWV group than in the low baPWV group
Grotenhuis HB et al. [43]	TA	crPWV, cfPWV	*n* = 7	crPWV and cfPWV higher than HC: 8.1 ± 1.8 vs. 6.4 ± 0.6 m/s, *p* = 0.03 und 8.3 ± 1.9 vs. 5.1 ± 0.8 m/s, *p* < 0.01, respectively
Ng WF et al. [44]	TA	PWV and AIx	*n* = 10	cfPWV higher in TA than in HC (*p* = 0.03)
Wang Z et al. [45]	TA	baPWV	*n* = 67	baPWV higher than in HC (*p* < 0.001)
Wang X et al. [46]	TA	baPWV	*n* = 48	baPWV higher than in HC (17.0 ± 3.8 vs. 13.8 ± 3.0 m/s; *p* = 0.002)
He Y et al. [47]	TA	baPWV	*n* = 74	baPWV associated with CV events (OR: 1.132, 95%CI: 1.063–1.204, *p* < 0.001)
Schillaci G et al. [53]	PMR	Aortic PWV	*n* = 39	Aortic PWV higher than in HC (12.4 ± 4 vs. 10.2 ± 2 m/s, *p* < 0.01)
Pieringer H et al. [54]	PMR	AIx	*n* = 13	Non-significant Aix difference: patients vs. HC (28.5 (9.1%) vs. 24.7 (6.4%); *p* = 0.19)
Emamifar A et al. [55]	PMR and GCA	Aortic PWV and AIx	*n* = 77	No PWV differences between patients with different grades of CV risk (all; *p* > 0.05)
Ooyanagi R et al. [56]	KD	PWV and ABI	*n* = 90	When a cut-off was set as % of normal predicted PWV (%N PWV) ≥ 120%, and ABI ≤ 0.9, KD- history patients had higher PWV than HC
Cheung YF et al. [57]	KD	Carotid artery stiffness index and brPWV	*n* = 72	cIMT correlated positively with carotid artery stiffness index (r = 0.40, *p* = 0.001) and brPWV (r = 0.28, *p* = 0.016)
Cheung YF et al. [58]	KD	Carotid stiffness index	*n* = 51	Biomarker levels correlated positively with carotid IMT (*p* < 0.001 and *p* = 0.034, respectively), and stiffness index (*p* = 0.001 and *p* = 0.021)
Nakagawa R et al. [60]	KD	baPWV, CAVI	*n* = 201	baPWV significantly higher than in HC (913 ± 121 cm/s vs. 886 ± 135 cm/s, *p* = 0.04) CAVI not significantly different between the two groups (*p* = 0.9)
AlHuzaimi A et al. [61]	KD	Aortic PWV	*n* = 42	PWV higher than in HC (495 vs. 370 cm/s, *p* = 0.0008)
Oyamada J et al. [62]	KD	Echo	*n* = 75	Coronary aneurysms and left ventricular mass index were independently relevant to aortic stiffness index and aortic distensibility
Chen KY et al. [63]	KD	PWV, carotid distensibility, and diameter compliance	*n* = 60	Patients with coronary artery abnormalities had reduced carotid distensibility compared to controls (15.16% (95% CI 13.67–16.65) vs. 17.50 (95% CI 16.43–18.58), *p* = 0.02)
Yildiz M et al. [71]	GPA	Aortic PWV	N = 10	cfPWV higher in GPA than in HC (*p* = 0.04)
Slot MC et al. [72]	AAV	Aortic PWV	N = 78	cfPWV higher in AAV than in HC (9.80 ± 2.50 m/s vs. 8.72 ± 1.68; *p* = 0.04)
Wilde B et al. [73]	AAV	PWV, A. femoralis	N = 83	PWV in AAV vs. HC: 9.8 ± 2.8 vs. 9.0 ± 2.2, *p* = 0.4
Farrah TE et al. [74]	AVV	PMV and AIx	N = 64	PMV and AIx higher in AVV than HC. (PWV: 7.3 ± 1.3 vs. 6.4 ± 1.0 m/s (*p* = 0.016); AIx: 26% ± 11% vs. 20% ± 10% (*p* = 0.031))
Caldas CA et al. [85]	BD	PWV	N = 46	PWV higher than HC (8.48 ± 1.14 vs. 7.53 ± 1.40 m/s, *p* = 0.017).
Yıldırım A et al. [86]	BD	PWV	N = 30	PWV higher than HC (6.35 ± 1.05 vs. 5.75 ± 0.83; *p* = 0.017).
Zencirkiran Agus H et al. [87]	BD	cfPWV	N = 90	PWV higher than HC (9.57 ± 1.88 vs. 8.47 ± 1.13 m/s; *p* = 0.003)
Protogerou AD et al. [88]	BD	AIx and AoSI	N = 98	AIx, but not AoSI, was lower in steroid taking patients than in steroid-free patients and similar to controls (21 ± 14% vs. 12 ± 14%, *p* < 0.05)
Yilmaz S et al. [89]	BD	PWV	N = 96	PWV values were higher in patients with active BD than in patients with inactive BD (*p* < 0.05)
Alis D et al. [90]	BD	cIMT and SWE	N = 62	Mean right (3.72 ± 0.94 m/s) and left (3.57 ± 0.72 m/s) SWE higher than mean right (2.42 ± 0.49 m/s) and left (2.56 ± 0.49 m/s) in HC (*p* < 0.001 for both)
Rhee MY et al. [91]	BD	DC, β and Einc	N = 94	DC (23.10 ± 9.5 vs. 27.90 ± 10.14, *p* = 0.021), β (3.26 ± 0.45 vs. 3.04 ± 0.32, *p* = 0.007), and Einc (0.64 ± 0.33 vs. 0.49 ± 0.16, *p* = 0.008) higher than in HC
Celik G et al. [92]	BD	AIx and PWV	N = 156	BD patients with high AIx had higher PWV (5.91 ± 1.26 vs. 5.20 ± 0.60; *p* = 0.013)
Kayikçioğlu M et al. [93]	BD	EDVD of brachial artery	N = 95	EDVD significantly impaired in patients with BD compared with HC (11.4 +/− 6.3 vs. 20.4 +/− 9.1%, *p* = 0.001)

TA: Takayasu’s arteritis; PMR: polymyalgia rheumatica; GCA: giant cell arteritis; KD: Kawasaki’s disease; GPA: granulomatosis with polyangitis; AAV: ANCA associated vasculitis; BD: Behcet’s disease; cfPWV: carotid–femoral pulse wave velocity; AIx: augmentation index; HC: healthy controls; Ep: Peterson’s elastic modulus; YM: Young’s elastic modulus; baPWV: brachial–ankle PWV; CV: cardiovascular; crPWV: cardio-radial PWV; brPWV: brachio-radial PWV; OR: odds ratio; ABI: ankle–brachial index; IMT: intima-media thickness; CAVI: cardio-ankle vascular index; AoSI: aortic stiffness index; SWE: shear wave elastography; DC: distensibility coefficient; β: stiffness index, Einc: incremental elastic modulus: EDVD: endothelium-dependent vasodilatation.

## Data Availability

No new data were generated for this study.
